# Molecular Changes Following Induction of Hepatocellular Carcinoma by Diethylnitrosamine and Thioacetamide, and Subsequent Treatment with* Dioscorea membranacea* Extract

**DOI:** 10.7150/ijms.72987

**Published:** 2022-10-09

**Authors:** Vichununt Kerdput, Kritsakorn Kanjanapongkul, Arunporn Itharat, Ratchadaporn Pramong, Wouter H Lamers, Theodorus B. M. Hakvoort, Aldo Jongejan, Wisuit Pradidarcheep

**Affiliations:** 1 Department of Anatomy, Faculty of Medicine, Srinakharinwirot University, Bangkok 10110, Thailand.; 2 Department of Science, Mahidol University International College, Mahidol University, Nakhon Pathom 73170, Thailand.; 3 Department of Applied Thai Traditional Medicine, Faculty of Medicine, Thammasat University, Pathumthani 10120, Thailand.; 4 Tytgat Institute for Liver and Intestinal Research, Amsterdam University Medical Centers, Location AMC, 1105 BK Amsterdam, The Netherlands.; 5 Bioinformatics Laboratory, Department of Epidemiology and Data Science, Amsterdam University Medical Centers, Location AMC, University of Amsterdam, 1105 AZ Amsterdam, The Netherlands.

**Keywords:** *Dioscorea membranacea*, liver cancer, apoptosis, malondialdehyde, RNA sequencing

## Abstract

Hepatocellular carcinoma (HCC) is a primary liver cancer commonly found in adults. Previously, we showed the anticancer effects of Thai herbal plant extract, *Dioscorea membranacea* Pierre (DM), in HCC-bearing rats. In the present study, we further examined the proposed mechanism of DM, including apoptosis and antioxidant activity. Moreover, we used RNA sequencing (RNA-seq) to analyze molecular pathways in the rat model in which HCC was induced by diethylnitrosamine (DEN) and thioacetamide (TAA). The HCC-bearing rats were then treated with 40 mg/kg of DM for 8 weeks, after which experimental and control rats were sacrificed and liver tissues were collected. The RNA-seq data of DEN/TAA-treated rats exhibited upregulation of 16 hallmark pathways, including epithelial mesenchymal transition, inflammatory responses, and angiogenesis (*p*<0.01). DM extract expanded the Bax protein-positive pericentral zone in the tumor areas and decreased hepatic malondialdehyde levels, implying a decrease in lipid peroxidation in liver. However, DM treatment did not ameliorate the molecular pathways induced in DEN/TAA-treated livers. Our findings indicate that DM extract has antioxidant activity and exerts its pro-apoptotic effect on rat HCCs *in vivo* at the (post-)translational level.

## Introduction

Hepatocellular carcinoma (HCC) is the most common primary malignant tumor of the liver and the fourth most common cause of cancer-related mortality in humans 1. Animal models are well-established tools used to further our understanding of HCC pathogenesis and provide important data to screen for drugs with effective anticancer properties 2. Our recent research has focused on a rat model of HCC to search for such drugs in the resources of traditional Thai medicine 3. To induce hepatocellular cancer, we selected two chemical agents, diethylnitrosamine (DEN) and thioacetamide (TAA), which are an established initiator and promoter, respectively, of HCC in rats 4, 5. A single dose of 200 mg/kg DEN causes DNA mutations in hepatocytes 6. However, continued tumor-cell promotion is necessary to permit the descendants of an initiated cell to survive and expand 7. To bring about the irreversible progression step in the tumor cells, we have utilized different doses and durations of TAA treatment in rats previously treated with a single dose of DEN. TAA induces oxidative stress and is hepatotoxic in rats 5. We observed that TAA administration caused inflammation, fibrosis, cirrhosis with regenerative nodules, and HCC depending on the dose and the duration of treatment 8, 9. We tested 100-300 mg/kg TAA twice or thrice weekly for 4 weeks and found that 300 mg/kg of TAA thrice weekly for 4 consecutive weeks starting 2 weeks after administration of 200 mg/kg of the initiator DEN induces HCC in a very reproducible way 3, 10. Since 90% of HCC cases arise from cirrhosis 11-13, the DEN/TAA model appears to be an appropriate model for inducing hepatocellular tumors on a background of liver injury.

Our group confirmed the reproducible development of HCCs in outbred male Wistar rats with a single injection of 200 mg/kg DEN both macro- and microscopically. Cirrhosis is the end-stage of liver fibrosis due to chronic liver injury and is characterized by deformation of the liver parenchyma, which acquires a nodular structure separated by fibrous septae 14. We further observed that the architectural arrangement of the cells forming the nodules changes into that of 3-4 layers thick hepatic cell cords without sinusoidal channels in between adjacent cell cords. Based on the histopathological analyses, we have concluded that, in addition to fibrosis and cirrhosis, angiogenesis 15, oxidative stress 16, and inhibition of apoptosis 17 are involved in the development and growth of the tumors, but the underlying mechanism remained unclear.

A fairly recent approach to better understand tumor development and progression relies on analysis of the cell's entire transcriptome. The comparison of spontaneous and chemically-induced liver tumors revealed that the mutational profile of a liver tumor induced by a single, relatively low dose of DEN (20 mg/kg) to preweaning male mice of a tumor-prone strain 18 produces less heterogenous tumors than develop spontaneously in the same strain 19, but whether this also applies to the more often used outbred rat strains is not known. Earlier studies have shown that DEN-induced tumors probably develops from mutated cells that have escaped p53-dependent cell removal 20. With respect to the cirrhotic DEN/TAA animal model only one such analysis is available 21. This relatively small microarray study demonstrated upregulation of extracellular-matrix deposition-associated genes. In the present study, therefore, we used RNA sequencing of our DEN/TAA rat model of hepatocarcinogenesis to obtain such information.

The standard treatment of human advanced HCC is the multi-kinase inhibitor Sorafenib (Nexavar; Bayer Pharmaceuticals) 22. This compound temporarily blocks HCC growth 23, also in our DEN/TAA rat model 3, but is associated with severe side effects 24. We have developed an interest in the alleged beneficial effects of Thai traditional medicines on the progression of HCC. Of these, we have recently tested Benja-ummarit 3 and *Dioscorea membranacea* Pierre (DM) extract 10 in our rat model, and have shown them to slow down cancer progression. In the present study, we aimed to investigate if the effect of DM extract on the progression of tumor growth in the DEN/TAA rat model was identifiable at the molecular level. We have, therefore, determined the mRNA profile in the livers of tumor-bearing and control rats that were either treated or not treated with DM extract. The samples were taken from rats in a study we recently reported 10. We opted to profile the mRNA expression in rats treated with DM extract because we deemed the effects of that extract most promising.

## Methods

### Preparation of *D. membranacea* (DM) extract

Dried ground plant material of DM (100 g) was percolated with 95% ethanol and evaporated to dryness under reduced pressure to prepare the plant extract stock 25. The extract quality was comparable to that established in a recent study 10.

### Animal experimental design

Male Wistar rats (*Rattus norvegicus*) weighing 200-250 g were obtained from the National Laboratory Animal Center, Mahidol University, Thailand**.** The rats were acclimatized for 2 weeks before experimentation.

The HCC groups were subjected to a single intraperitoneal dose of 200 mg/kg diethylnitrosamine (DEN; Sigma-Aldrich, St**.** Louis, MO, USA). After two weeks, the rats were intraperitoneally administered 300 mg/kg of thioacetamide (TAA; Sigma-Aldrich, St. Louis, MO, USA) three times per week for 4 consecutive weeks, and then left for a further 2 weeks without any treatment. After HCC induction, the normal control (n=6) and untreated-HCC groups (n=6) received vehicle alone (propylene glycol:tween80:water 4:1:4 v/v) for 8 weeks. The treatment groups received 40 mg**/**kg of DM extract (DM40) daily by oral gavage for 8 weeks. In total, the experiment lasted 16 weeks. At the end of the experiment, rats were anesthetized under 45 mg/kg Nembutal prior to decapitation with a rodent guillotine. Liver tissues were collected for RNA sequencing, immunohistochemistry, Western blot, and MDA analysis.

### RNA isolation and sequence analysis

RNA was extracted from snap-frozen rat liver after homogenization of the samples in TRIzol reagent according to the manufacturer's instructions (Invitrogen, Carlsbad, CA, USA). RNA quality was assured with the Bioanalyzer (Agilent, Santa Clara, USA), and only samples with RIN scores >8 were used. mRNA was isolated and converted into cDNA with the KAPA mRNA HyperPrep Kit (Roche). cDNA libraries were prepared for sequencing on the HiSeq4000 at the Core Facility Genomics, Amsterdam University Medical Centers, in a 50 bp single-ended fashion to a depth of 40M per sample. The raw reads were checked for quality using FastQC (v0.11.8) and summarized in feature Counts MultiQC (v1.7) 26. Alignments to the *Rattus norvegicus* genome (Rnor v6.0) used STAR (v2.6.1a) and were annotated with Ensembl v95 27. Genes with more than 2 counts-per-million reads (CPM) in 3 or more of the samples were kept. For normalization the weighted trimmed mean of M-values (relative to the reference) was used (TMM (edgeR)) 28. Genes were reannotated using BiomaRt and Ensembl (v99). The count data was transformed to ^2^log-counts per million (logCPM) using “voom”, which estimates the mean-variance relationship. Differential expression was assessed by a Bayes moderated t-test, using the linear model framework from the Limma package 29. The Benjamini-Hochberg false discovery rate was used to correct for multiple testing of the resulting p-values. Homologene v68 (ftp://ftp.ncbi.nih.gov/pub/HomoloGene/) was used to map the Entrez Gene IDS from *R. norvegicus* to human to be able to perform a gene set enrichment analysis against the human Genesets from MSigDB v7.0 (collections H,C1,C2,C3,C5,C6,C7) (http://www.broadinstitute.org/gsea/msigdb/index.jsp). For this geneset enrichment analysis we used the CAMERA (http://nar.oxfordjournals.org/content/early/2012/05/24/nar.gks461.abstract) function of the Limma package, with inter.gene.corr=0.01. Analysis was performed using R v3.6.1 (https://www.r-project.org/) and Bioconductor v3.9 30. The RNA-seq data are available on Sequence Read Archive under the BioProject: PRJNA774039.

### Quantitative real-time PCR (RT-qPCR) analysis

We selected 8 genes that showed up-regulation in response to RNA-Seq (log2 fold change>1.8) for RT-qPCR validation. The RT-qPCR was performed in CFX96 Real-Time PCR Detection System (Bio-Rad, Hercules, CA, USA), using SsoAdvanced Universal SYBR Green Supermix with commercial PrimePCR primers (Bio-Rad, Hercules, CA, USA). All primers used in this study are shown in Table 1. Rat *Actb*, *Gapdh*, and *Hprt1* mRNA were used as the internal controls. The thermal cycler protocol consisted of 2 minutes of activation at 95°C followed by 40 cycles of denaturation at 95°C for 5 seconds and then annealing/extension at 60°C for 30 seconds. The mRNA expression analysis was performed by Bio-Rad CFX manager software version 1.3.1 (Hercules, C. The quantification of relative mRNA expression was calculated using the 2^-ΔΔCT^ (Livak) method 31.

### Immunohistochemistry

Liver sections were deparaffinized and rehydrated in a graded series of ethanol. Immunostaining was performed using ImmunoCruz® rabbit ABC Staining System (sc-2018, Santa Cruz Biotechnology Inc., Santa Cruz, CA, USA). Antigen retrieval was performed by heating the liver samples in an autoclave for 10 minutes at 120°C in 10mM sodium citrate. After cooling down, the sections were incubated in 3% hydrogen peroxide in methanol for 20 minutes to quench endogenous peroxidase activity and washed with phosphate-buffered saline (PBS) twice. The sections were then incubated with 1.5% blocking serum in PBS for 1.5 hours in a humidified chamber. The sections were incubated with rabbit anti-Bax antibody (1:250, ab32503, Abcam, Cambridge, MA, USA) and rabbit anti-Bcl-2 antibody (1:100, ab196495, Abcam, Cambridge, MA, USA) diluted in blocking serum overnight at 4°C in a humidified chamber. The sections were washed with PBS, followed by incubation for 1.5 hours with biotinylated secondary antibody. After washing with PBS, the sections were incubated with avidin and biotinylated horseradish peroxidase (AB reagents) for 30 minutes, washed with PBS, and further incubated with peroxidase substrate. After washing with PBS, counter staining was performed by incubating the sections in Mayer's hematoxylin (Bio-Optica, Milano, Italy) for 1 minute. After washing in distilled water, the sections were dehydrated and cleared in xylene. The sections were mounted with Permount, then slides were photographed under a light microscope (Olympus, Tokyo, Japan).

### Western blot analysis

Forty milligram of liver tissue was mixed with 350 µL of RIPA (Radioimmunoprecipitation) Lysis Buffer System (Santa Cruz Biotechnology, CA, USA), sonicated for 10 seconds, and centrifuged for 15 minutes at 12,000 rpm at 4 °C. The protein concentration in the supernatant was determined with the Bradford assay (Bio-Rad, Hercules, CA, USA), with bovine serum albumin as protein standard. After loading the gels with 50 µg of protein, proteins were separated by 12.5% sodium dodecyl sulfate-polyacrylamide gel electrophoresis (SDS-PAGE), then transferred to polyvinylidene difluoride (PVDF) membranes. Membranes were blocked for non-specific binding by incubating for 1 hour in blocking solution (5% skim milk powder in Tris-buffered saline containing 0.1% Tween-20 (TBST), pH 6.8) at room temperature. After blocking, the membranes were incubated in rabbit anti-Bax antibody (1:5,000, ab32503) or rabbit anti-Bcl-2 antibody (1:5,000, ab196495) diluted in 5% skim milk powder in TBST pH 6.8 for 18 hours at 4 °C. The membranes were washed with TBST buffer and incubated in anti-rabbit IgG, linked to horseradish peroxidase (1:5,000, #7074, Cell Signaling Technology, Danvers, MA, USA) diluted in 3% skim milk at room temperature for 1 hour. After washing with TBST buffer, protein bands were developed by enhanced chemiluminescence (ECL) using Clarity Western ECL Substrate (#1705061, Bio-Rad, Hercules, CA, USA) and imaged on a Syngene (UK) gel documentation system. The protein bands were quantified by measuring the density of each band with the Scion Image program (National Institutes of Health, Bethesda, MD). GAPDH (1:5,000, Invitrogen, Carlsbad, CA, USA) was used as a loading control.

### Malondialdehyde (MDA) assay

Lipid peroxidation was determined by assaying malondialdehyde (MDA) with thiobarbituric acid (TBA) using a commercial kit (MAK085, Sigma**-**Aldrich, St. Louis, MO, USA). Briefly, 20 mg of liver samples were sonicated in 300 µL of MDA lysis buffer and centrifuged at 13,000 g for 10 minutes at 4 °C. 200 µL of supernatant was collected, and 0.6 mL of the TBA solution was added to form the MDA**-**TBA adduct. Absorbance was measured at 532 nm with a spectrophotometer. The concentration of MDA present in the samples was determined from a calibration curve of the MDA standard as described in the manufacturer's instruction.

### Statistical analysis

Data were analyzed using a one-way analysis of variance with a Bonferroni post-hoc test (GraphPad Prism 7.0).

## Results

### Fraction of genes that is affected by the tumor production protocol

More than 15,000 different mRNAs that qualified (>2 counts-per-million reads (CPM) in ≥3 samples) were identified. Of these, 655 (~4%) were differentially expressed (P_adj_<0.01) between HCC and control samples. 466 mRNAs were upregulated (71%) and 190 downregulated in the tumor tissue. All data were subjected to pathway enrichment analysis, using the Molecular Signatures Database (MSigDB) 32. Using just the relatively non-redundant collection of “hallmark” gene sets in MSigDB, we observed that 16 processes were significantly upregulated with *p*<0.01 and 10 additional ones with *p*<0.05 (Table 2), whereas none of the processes analyzed was significantly downregulated. Genes involved in the establishment of cell structure, inflammatory responses, vessel formation, and metabolic, executive, and signaling pathways were regulated, whereas DNA damage and proliferation processes were hardly affected. The strong upregulation of the expression of genes involved in epithelial-mesenchymal transition (EMT) may relate to hepatocellular dedifferentiation in the tumors 33,34. The identification of highly regulated genes will allow a choice of marker genes to follow the above-mentioned processes in our rat hepatoma model. Apparently, inflammation, fibrosis, angiogenesis, and also metabolism are regulated by exposure to DEN/TAA and tumor development.

**Figure 1:**
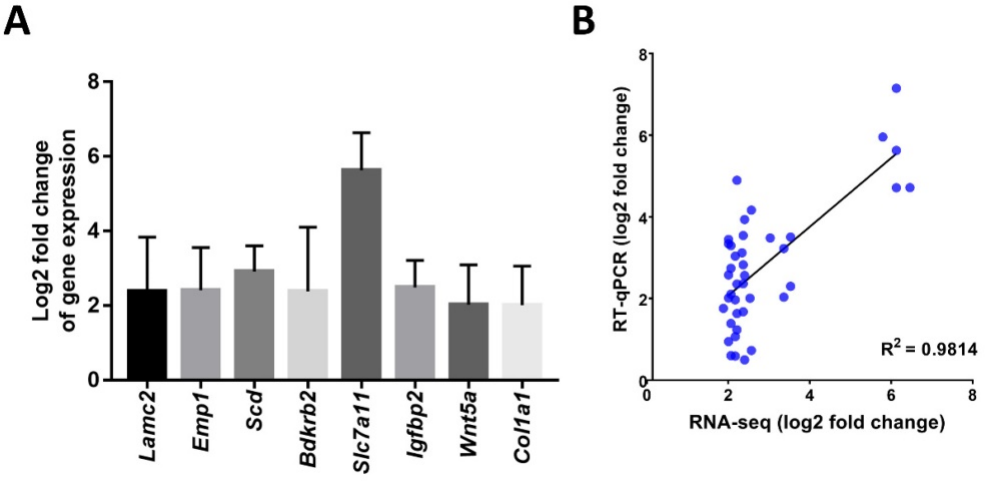
** A)** Log2 fold changes (Control vs. HCC) of representative genes measured by RT-qPCR; **B)** Correlation of gene expression log2 fold change obtained by RT-qPCR and RNA-seq. All RT-qPCR data was collected from five biological replicates.

The mRNAseq data were validated with RT-qPCR assays. Comparison of both assays revealed that the log2 fold change of 8 genes which showed upregulation in the mRNAseq assays of the HCC group compared to the control group (*Lamc2*, *Emp1*, *Scd*, *Bdkrb2*, *Slc7a11*, *Igfbp2*, *Wnt5a*, and *Col1a1*) were also upregulated in the RT-qPCR assays (Figure 1A). The Pearson correlation coefficient of this comparison was 0.98 (*p*<0.001; Figure 1B).

### DM extract does not change the expression of hepatoma-associated genes

We also analyzed gene expression in livers of HCC rats treated with DM extract. The number of analyzed animals and the analysis protocol were the same as those for the non-treated rat groups. 259 genes were differentially expressed at (*p*<0.01). Unexpectedly, we found that the expression of none of these 259 genes was significantly regulated (P_adj_ <0.01) by the DM extract in the tumor-bearing groups. The expression of only 2 genes was differentially expressed at P_adj_ <0.05 (glutathione hydrolase 1 (*Ggt1*) and stearoyl-CoA desaturase 2 (*Scd2*) mRNA (*p*<0.01) and the downregulation of epithelial membrane protein-1 (*Emp1*; *p*<0.05). In the non-tumor-bearing control group, not a single gene was differentially affected by DM extract.

**Figure 2:**
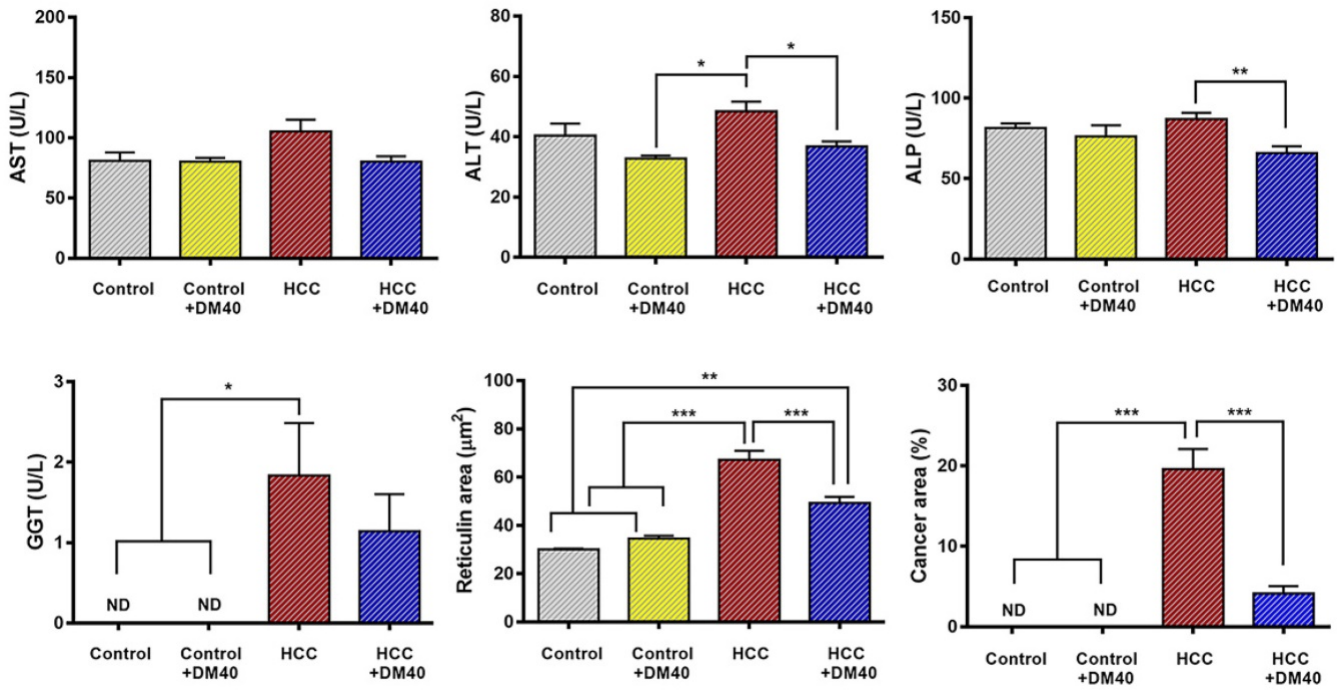
Serum levels of aspartate aminotransferase (AST), alanine aminotransferase (ALT), alkaline phosphatase (ALP), g-glutamyl transferase (GGT) activities, and the area of reticulin cancer (*p<0.05, **p<0.01, ***p<0.001, ND: non-detectable) in the four experimental groups of rats (control, control treated with DM40, HCC, HCC treated with DM40). The data represent a subsample of those described in reference 10 to evaluate the effect of DM40 in a 2*2 comparison.

Compared to control rats, the increase in circulating liver enzymes in HCC-bearing rats was modest (Figure 2). Confirming our earlier data 10, DM extract nevertheless, reduced plasma ALT and ALP levels significantly in the HCC-bearing rats. This finding suggests that DM has a small adjuvant effect. Furthermore, DM treatment of HCC-bearing rats significantly decreased the area that stained for reticulin and that of cancer cells in sections (Figure 2). These findings indicate that the DM extract was effective in reducing hepatocyte injury.

### DM can elevate key proteins of the apoptotic pathway in HCC-bearing rats

**Figure 3:**
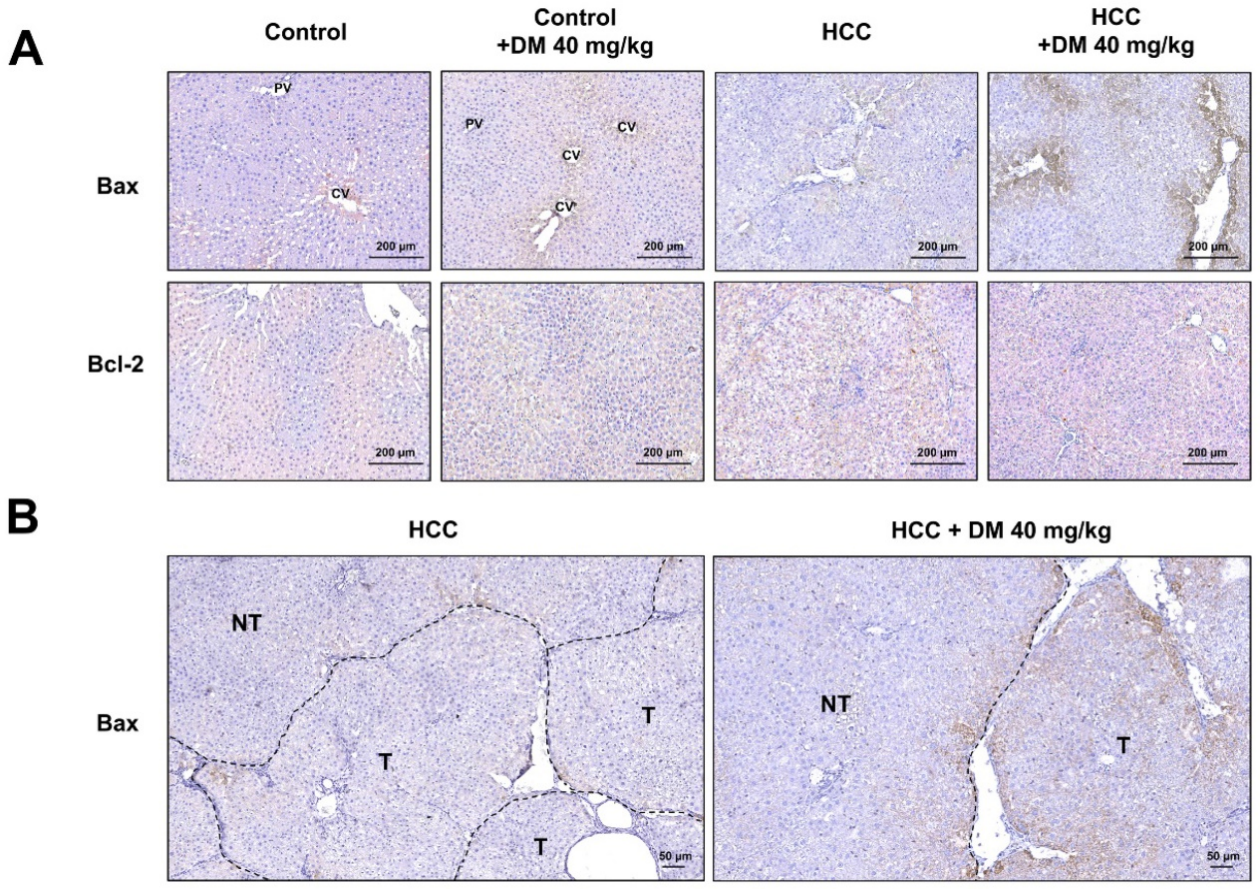
** A)** Immunohistochemical staining of BAX and BCL-2 in the livers of the four experimental groups of rats; **B)** The expression of BAX in tumorous (T) and non-tumorous (NT) areas of HCC and DM-treated HCC rat tissues. CV; central vein, PV; portal vein.

**Figure 4:**
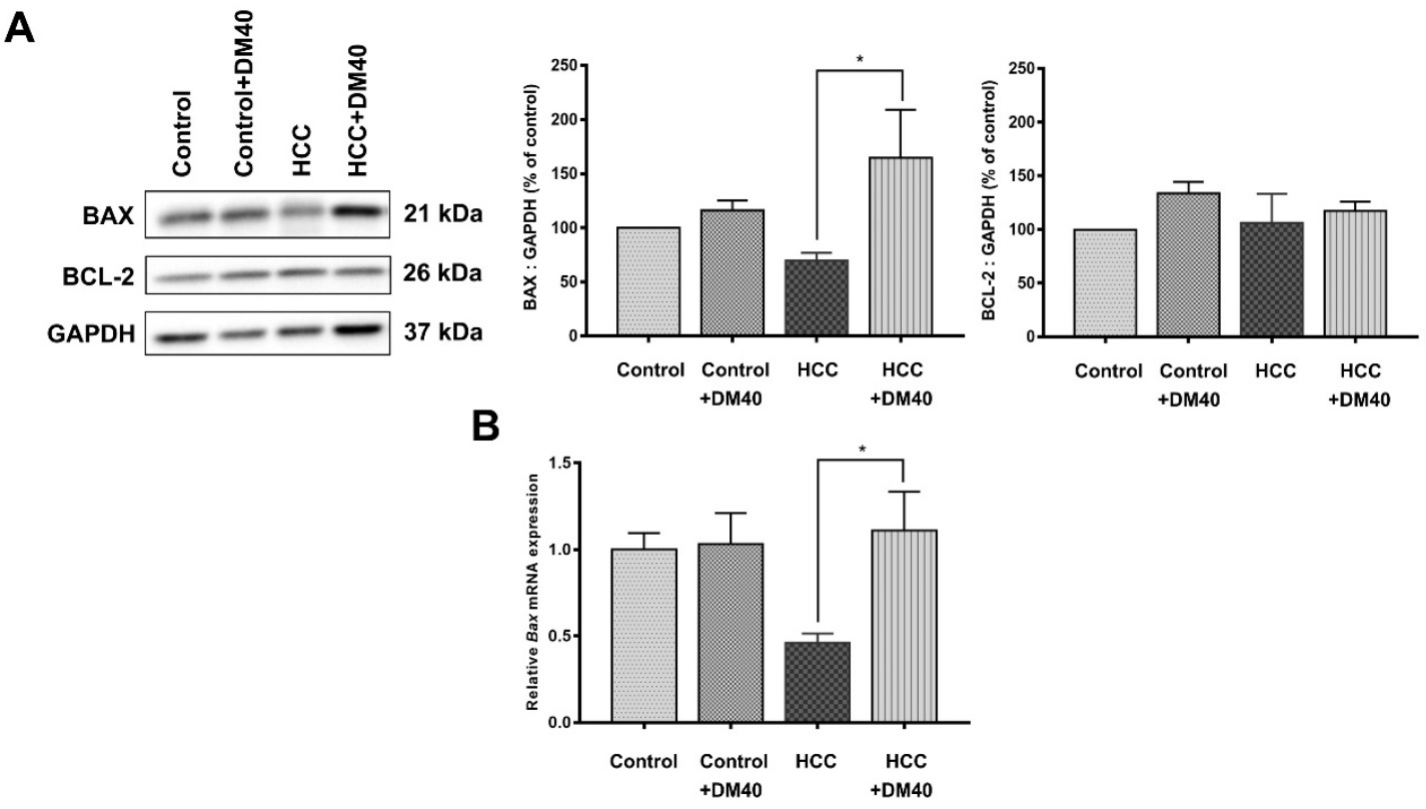
** A)** The expression of BAX and BCL-2 protein as determined by Western blot analysis; **B)** *Bax* mRNA expression as determined by RT-qPCR (*p<0.05).

The apoptosis pathway is upregulated in DEN/TAA-induced tumors (Table 2; *p*<0.009). DM enhances apoptosis in liver cancers of DEN/TAA-treated rats, as shown by the DNA fragmentation TUNEL assay reported in our recent study 10. To investigate if this pro-apoptotic effect of DM is caused by increased expression of key proteins in the apoptotic pathway, we stained sections immunohistochemically, and quantified Western blots for the presence of BAX and BCL-2 proteins*.* BAX expression in normal non-treated-and DM-treated rats was immunopositive only in a few layers of hepatocytes surrounding the central veins, whereas BCL-2 was present diffusely throughout the liver tissue (Figure 3A). In non-treated HCC-bearing rats, apart from the pericentral area, the staining of BAX was less intense and found at the peripheral part of the tumor nodule. The administration of DM extract enhanced the staining of BAX in tumor and peritumoral areas. However, compared with the tumor areas, BAX immunostaining was quite minimal in non-tumorous areas of HCC or DM-treated HCC rat livers (Figure 3B). Accordingly, the concentration of BAX in DM-treated HCC-bearing rat liver was significantly increased compared to that in non-treated HCC liver (*p*<0.05; Figure 4A). The distribution and staining intensity of BCL-2 was similar in all groups (Figure 3A). In agreement, the BCL-2 content in liver extracts as measured with Western blots was similar in all groups (Figure 4A). In addition, the RT-qPCR assay demonstrated that *Bax* mRNA expression was upregulated in DM-treated HCC-bearing rats compared to the non-treated HCC group (*p*<0.05) (Figure 4B). These findings show that DM extract regulates at least some steps of the apoptotic pathway in HCCs at the protein level, whereas it did not affect this pathway in healthy livers.

### DM reduces lipid peroxidation in HCC-bearing rats

**Figure 5:**
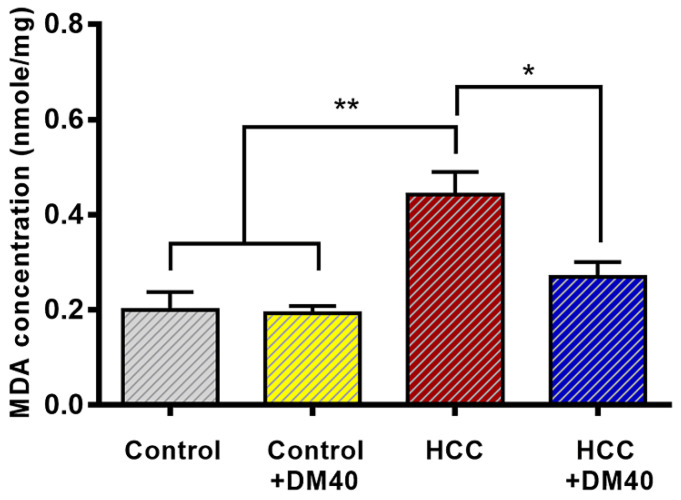
MDA concentrations in the livers of the respective rat groups. (*p<0.05, **p<0.01).

The MDA assay, which determines lipid peroxidation, was used to measure the antioxidant properties of DM extract. It showed significantly increased MDA concentrations in the HCC group compared to the control group (*p*<0.01) and reduced MDA concentrations to ~60% of controls in the DM-treated groups (*p*<0.05; Figure 5).

## Discussion

In this study, we showed that 16 Hallmark pathways were very significantly upregulated (*p*<0.01) and an additional 10 pathways slightly less significantly (*p*<0.05) in the DEN/TAA HCC model. The outcome of the present cell-wide mRNAseq analysis corresponds largely with the earlier reported 14 “main upregulated” genes in the study of Romualdo et al (Supplementary Table 1; 21). The pathways establish a well-defined expression profile for this liver tumor model that we then used to study the effects of interventions with Thai traditional medicines 3, 10. Our earlier 10 and the present studies show that DM extract improves the histopathology of the tumors (smaller cancer areas, reduction of reticulin and glypican3 staining), limits parenchymal damage (lower serum transaminase and alkaline phosphate activity) 10, increases the hepatic protein content of BAX (Figure 4), and causes less lipid peroxidation (Figure 5). Unexpectedly, however, the mRNA expression profile of the DM-treated HCC rats was not different from that of the non-treated tumor-bearing rats. In aggregate, these data therefore suggest that the reported effects of DM extract are mediated by processes at the (post-)translational level.

The DEN/TAA model of rodent liver cancer is widely used to study potential treatment options for hepatocellular carcinoma 4, 5, 21, but the protocols used vary considerably. DEN is a powerful genotoxic agent 15, 35, which is often administered only once to preweaning male mice at a dose of 5-25 mg/kg 19, 36. Male rats are usually treated later, at 5-9 weeks of age, with 10-200 mg/kg. Furthermore, DEN administration is repeated several times in some protocols 37, 38. After a low dose of DEN, mice do not develop fibrosis 39 or HCC 40. Inflammation and fibrosis are more prominent if DEN is administered at intermediate doses (5-90 mg/kg). Under such a regimen, mice develop premalignant lesions after 24 weeks, and HCC after ~1 year 36, 40. If DEN is repeatedly administered, the effects are more severe 37, 38.

Human HCC is present 5 to 6-fold more often in cirrhotic than in non-cirrhotic livers 41, 42. Given the moderately profibrotic activity of DEN at intermediate doses, a strong pro-fibrotic hepatotoxin, such as CCl_4_ or TAA, is often added to the DEN protocol as a promoting agent. Such a two-stage model assumes that the induction of the tumor is caused by a genotoxic compound, such as DEN, and that a promoting compound without genotoxic activity enhances tumor formation 40. We used TAA as promoting agent. TAA induces hepatotoxicity after biotransformation by CYP450 enzymes and flavin monoxygenase to TAA-S-oxide, which modifies amine-lipids and proteins 43. When treatment is chronic, TAA is administered at doses between 25 and 280 mg/kg 44, 45. Low doses cause apoptosis, whereas doses of ≥150 mg/kg cause necrosis of pericentral hepatocytes within a day 46, 47. Chronic administration of TAA causes hepatitis at 6 weeks, advanced fibrosis at 12 weeks, and macronodular cirrhosis at 18 weeks 48.

The respective roles of DEN as mutagenic compound and TAA as promoting compound raise the question whether the genomic signatures that we found reflect either one or both compounds. The mutational profiles of DEN-treated mice were similar between different mice and between dysplastic and tumorous nodules in the same mouse, implying reproducible, stable mutations 19. In contrast to human HCC, DEN mutations in rodents rarely involve TP53 and CTNNB1 35, 49, explaining the typically decreased expression of glutamine synthetase in DEN-treated animals 37. DEN does mutate, however, *Braf*, *Hras*, and *Egfr* preferentially 19, 35. Unfortunately, the mutational profile of TAA has been characterized in less detail 50. Nevertheless, it seems possible to recognize typical DEN-induced pathways, such as “estrogen_response_early and _late” and “androgen_response” (Table 2). This hormonal response imprint corresponds with the much higher sensitivity of male than female rodents to the induction of mutations by DEN 18, 40, 51, which may relate, in turn, to sex-dependent differences in growth hormone secretion by hepatocytes 52 and IL-6 production in Kupffer cells 18. Such a sex difference is also found in human HCC 41. The effect on the “coagulation” pathway appears to correspond with TAA-related thrombocytopenia 53, while the “apical_junction” and “apical_surface pathways appear to reflect TAA-induced bile duct epithelial damage 54, 55. The pro-fibrotic effects of TAA are well-studied and seem to be a sequel of its cytotoxicity which peaks between 24 and 48 hours after administration 44, 47. DEN has, in addition to mutagenic, also pro-inflammatory effects that appear secondary to its hepatotoxicity 18, 36, but these effects are probably limited, because the compound is usually administered only once. TAA, in contrast, is repeatedly administered, which induces cycles of cell death, regeneration and ECM deposition, and well-developed fibrosis at ~12 weeks 48. Accordingly, human HCCs usually develop in the context of chronic liver disease 51. Given the fact that 4- to 5-fold more “spontaneous” human and mouse HCCs develop in a cirrhotic than in a non-cirrhotic liver 40, 42, 56, hallmark pathways such as “epithelial_mesenchymal_transition”, “angiogenesis”, “TGF_β_signaling”, therefore reflect mostly TAA-induced fibrosis. From this brief overview, we conclude that the contributions of both DEN and TAA remain identifiable in rodent HCCs.

Apart from the molecular aspects of hepatocarcinogenesis, the metabolic sequels of the genomic alterations also have their effect at the posttranslational level. A recent study in human 57 identified metabolites that were associated specifically with HCCs in a fibrotic or cirrhotic environment and that predominated in a pre-fibrotic environment. The majority of these changes in metabolite concentration are in all likelihood secondary to changes at the mRNA level, but choline, one of the metabolites, causes or aggravates fatty liver, while another, glutamine, is a key interorgan transporter of amino groups and a precursor to neurotransmitters. We ourselves observed effects on lipid peroxidation (Figure 5).

The RNA sequencing data were strictly filtered so that differences between conditions were only included if that difference exceeded a 2.6-fold change. The analysis of the filtered data revealed that the liver tumors developed a clear molecular profile under our experimental conditions. Since we did observe clear-cut effects of DM extract on tumor size and environment, and less parenchymal damage (lower serum enzymes), the beneficial effects of DM extract must be looked for at the (post-)translational level. We hypothesize that DM, not being a pure substance, has several small but additive, rather than one large beneficial effect on liver tumors. Given the findings, it is well possible that some, or even the majority, of the effects of DM extract are mediated by (post-)translational mechanisms.

In conclusion, the present and our earlier study 10 showed that DM extract reduces the cancer volume in livers, promotes apoptosis and antagonizes oxidative stress and hepatocellular enzyme leakage, but does not mediate these effects via changes at the transcriptional level.

## Supplementary Material

Supplementary table.

## Figures and Tables

**Table 1:** PrimePCR primers designed for SYBR^®^ Green gene expression

Gene symbol	Unique Assay ID	GenBank accession No
*Actb*	qRnoCID0056984	NM_031144
*Gapdh*	qRnoCID0057018	NM_017008
*Hprt1*	qRnoCED0057020	NM_012583
*Igfbp2*	qRnoCID0008306	NM_013122
*Lamc2*	qRnoCID0003848	Not Available
*Scd*	qRnoCED0007508	NM_031841
*Col1a1*	qRnoCED0007857	NM_053304
*Bdkrb2*	qRnoCID0003331	NM_173100
*Emp1*	qRnoCID0052105	NM_012843
*Slc7a11*	qRnoCID0005153	Not Available
*Wnt5a*	qRnoCID0006042	Not Available
*Bax*	qRnoCED0002625	Not Available

**Table 2:** Functional processes that are affected by the DEN/TAA tumor-production protocol

Hallmark gene sets	N	*p*-value	FDR
Epithelial_mesenchymal_transition	160	1.42E-08	2.57E-05
Allograft_rejection	169	1.13E-05	2.16E-03
Kras_signaling_up	159	6.85E-05	6.19E-03
Estrogen_response_late	165	1.25E-04	8.93E-03
Mtorc1_signaling	185	2.62E-04	1.40E-02
Myc_targets_v1	178	4.02E-04	1.76E-02
Cholesterol_homeostasis	72	4.50E-04	1.84E-02
Angiogenesis	35	2.27E-03	4.74E-02
112_Stat5_signaling	170	2.34E-03	4.82E-02
lnflammatory_response	165	2.76E-03	5.34E-02
Myogenesis	143	4.05E-03	6.55E-02
Coagulation	116	4.29E-03	6.72E-02
Glycolysis	170	4.69E-03	7.10E-02
Tgfl3_signaling	51	5.44E-03	7.64E-02
Complement	166	6.11E-03	8.16E-02
Apoptosis	142	7.64E-03	9.08E-02
lnterferon_y_response	180	1.06E-02	1.08E-01
Androgen_ response	93	1.23E-02	1.17E-01
Apical_junction	162	1.25E-02	1.17E-01
Protein secretion	85	1.62E-02	1.32E-01
Apical_surface	38	1.83E-02	1.41E-01
Estrogen_response_early	168	2.14E-02	1.53E-01
lnterferon_a_response	88	2.57E-02	1.67E-01
Reactive_oxygen_species_pathway	46	2.60E-02	1.68E-01
Tnfa_signaling_via_NFKB	177	2.84E-02	1.75E-01
Hypoxia	164	3.47E-02	1.94E-01

Pathway enrichment analysis was performed and found to be significantly changed in the shown Hallmark gene sets. The number of founding genes (N) in the Hallmark sets is shown, because a larger number tends to result in a more significant *p*-value. The last column shows the calculated false discovery rate (FDR) of the findings.

## Abbreviations

ALP: alkaline phosphatase
ALT: alanine aminotransferase
AST: aspartate aminotransferase
*Bax*/BAX: Bcl-2-associated X protein
*Bcl-2*/BCL-2: b-cell lymphoma 2
CCI_4_: carbon tetrachloride
CYP450: cytochrome P450
DEN: diethylnitrosamine
DM: *Dioscorea membranacea*
*Emp1*: epithelial membrane protein-1
EMT: epithelial-mesenchymal transition
GGT: γ-glutamyltransferase
Ggt1: glutathione hydrolase 1
HCC: hepatocellular carcinoma
IL-6: Interleukin 6
MDA: malondialdehyde
MSigDB: molecular signatures database
PBS: phosphate-buffered saline
RT-qPCR: quantitative real-time polymerase chain reaction
*Scd2*: stearoyl-CoA desaturase 2
SDS-PAGE: sodium dodecyl sulfate-polyacrylamide gel electrophoresis
TAA: thioacetamide
TBA: thiobarbituric acid
TUNEL: terminal deoxynucleotidyl transferase dUTP nick end labeling
