# Supplementary material for: Molecular Changes Following Induction of Hepatocellular Carcinoma by Diethylnitrosamine and Thioacetamide, and Subsequent Treatment with Dioscorea membranacea Extract
Source: Int J Med Sci. 2022 Oct 9;19(12):1806–15. doi: 10.7150/ijms.72987 (PMC9608040; doi:10.7150/ijms.72987)
Supplement: Supplementary file 1 — Supplementary table. [file ijmsv19p1806s1.pdf]

Supplementary Table 1. Comparison of genes responding to DEN/TAA in Romualdo et al and Kerdput et al.

Gene symbol	Kerdput et al	Romualdo et al
	Log2 fold change	Log2 fold change
Col1a1	1.86	1.95
Col1a2	NI*	2.41
Anxa2	1.43	3.60
Dcn	0.56	1.79
Lgals3bp	1.53	2.19
Timp1	0.34	2.55
Timp2	0.88	1.59
Gstp1	4.95	6.89
Gstp2	NI*	5.98
Lsp1	0.85	1.74
Ccl21b	NI*	2.49
C1qa	0.19	1.60
C1qb	0.34	1.64
Ctse	1.05	2.48
Gpx1	-0.29	-1.98
Cat	-0.20	-1.89
Gstm3	NI*	-2.72

NI*: not identified
